# Supplementary material for: Mycotoxin Exposure during the First 1000 Days of Life and Its Impact on Children’s Health: A Clinical Overview
Source: Toxins (Basel). 2022 Mar 4;14(3):189. doi: 10.3390/toxins14030189 (PMC8955462; doi:10.3390/toxins14030189)
Supplement: Supplementary file 1 [file toxins-14-00189-s001.zip › toxins-1580860-supplementary.pdf]

toxins

MDPI

Review

Mycotoxin Exposure during the First 1000 Days of Life and Its Impact on Children's Health: A Clinical Overview

Paula Alvito and Luís Pereira-da-Silva

Table S1. Studies on pregnancy outcomes and maternal mycotoxin exposure in different continents (in alphabetic order of the first author).

	Ref.
Abdulrazzaq, Y.M. et al (2002). Fetal exposure to aflatoxins in the United Arab Emirates.	[48]
Abdulrazzaq, Y.M. et al (2004). Morbidity in neonates of mothers who have ingested aflatoxins.	[42]
Abulu, E.O. et al (1998) Preliminary investigation on aflatoxin in cord blood of jaundiced neonates.	[40]
Ahmed, H. et al (1995). Neonatal jaundice with reference to aflatoxins: An aetiological study in Zaria, northern Nigeria.	[43]
De Vries, H.R, et al (1989). Foetal and neonatal exposure to aflatoxins.	[37]
Jonsyn, F.E., et al (1995). Human fetal exposure to ochratoxin A and aflatoxins.	[38]
Kyei, N.N.A., et al (2020). Maternal mycotoxin exposure and adverse pregnancy outcomes: A systematic review.	[11]
Lauer, J.M. et al (2018). Maternal aflatoxin exposure during pregnancy and adverse birth outcomes in Uganda.	[47]
Moodley, D.M.J. et al (2001). An aetiological role in pre-eclampsia.	[44]
Shuaib, F.M.B. et al (2010). Association between birth outcomes and aflatoxin B1 biomarker blood levels in pregnant women in Kumasi, Ghana.	[46]
Sodeinde, O. Et al (1995). Neonatal jaundice, aflatoxins and naphthols: Report of a study in Ibadan, Nigeria.	[41]
Turner, P.C. et al (2007). Aflatoxin exposure in utero causes growth faltering in Gambian infants.	[39]

Table S2. Studies on AFM_1 and OTA highest concentration values in breast milk in different continentes (in alphabetic order of the first author).

	Ref.
Abdulrazzaq, Y.M. et al (2002). Fetal exposure to aflatoxins in the United Arab Emirates.	[48]
Braun, D., et al (2019). Mycotoxin-mixture assessment in mother-infant pairs in Nigeria: From mothers' meal to infants' urine.	[62]
Cantú-Cornelio F. et al (2016). Occurrence and factors associated with the presence of aflatoxin M1 in breast milk samples of nursing mothers in central Mexico.	[60]
Coppa, C.C.; et al (2019). The occurrence of mycotoxins in breast milk, fruit products and cereal-based infant formula: A review.	[49]
Hernández, M., et al (2021). Evaluation of mycotoxins in infant breast milk and infant food, reviewing the literature data.	[22]
Ishikawa A.T. et al (2016) Exposure assessment of infants to Aflatoxin M_1 through consumption of breast milk and infant powdered milk in Brazil.	[61]
Kamali, A. et al (2017). Detection of ochratoxin A in human breast milk in Jiroft city, south of Iran.	[56]
Memis, E.Y. et al (2021). Mycotoxin carry-over in breast milk and weight of infant in exclusively breastfed infants.	[63]
Muñoz, K. et al (2013). Exposure of infants to ochratoxin A with breast milk.	[57]

Ortiz, J. et al (2018). Multiple mycotoxin exposure of infants and young children via breastfeeding and complementary/weaning foods consumption in Ecuadorian highlands.	[52]
Peraica, M., et al (2014). Mycotoxicoses in children.	[16]
Radonić, J.R. et al (2016). Occurrence of aflatoxin M1 in human milk samples in Vojvodina, Serbia: Estimation of average daily intake by babies.	[51]
Samiee F. et al (2020). An assessment of the occurrence and nutritional factors associated with aflatoxin M1, ochratoxin A, and zearalenone in the breast milk of nursing mothers in Hamadan, Iran.	[64]
Tomerak, R.H. et al (2011). Assessment of exposure of Egyptian infants to aflatoxin M1 through breast milk.	[50]
Zinedine, A., et al (2021). Aflatoxin M1 in Africa: Exposure assessment, regulations, and prevention strategies - A review	[59]

Table S3. Studies assessing mycotoxin contamination in infant formulas, cereal-based products for infants, and fruit-based products for infants (in alphabetic order of the first author).

	Ref.
Alvito, P.C et al (2010). Occurrence of aflatoxins and ochratoxin A in baby foods in Portugal.	[15]
Assunção, R. et al (2018). Portuguese children dietary exposure to multiple mycotoxins—An overview of risk assessment under MYCOMIX project.	[69]
Bogalho, F.; et al (2018). Exposure assessment of Portuguese infants to aflatoxin M1 in breast milk and maternal social-demographical and food consumption determinants.	[68]
Bonerba, E et al (2010). Assessment of Dietary Intake of Patulin from Baby Foods.	[70]
Coppa, C.C (2019) et al. The occurrence of mycotoxins in breast milk, fruit products and cereal-based infant formula: A review.	[49]
Commission. Commission Regulation (EC) No 1881/2006 of 19 December 2006, Setting Maximum Levels for Certain Contaminants in Foodstuffs; European Union: Brussels, Belgium, Off. J. Eur. Union 2006, 364, 5-24.	[53]
Commission. Commission Regulation (EC) No 165/2010 of 26 February 2010, Amending Regulation (EC) No 1881/2006setting Maximum Levels for Certain Contaminants in Foodstuffs as Regards Aflatoxins. Off. J. Eur. Union 2009, 50, 8–12	[54]
Hernández, M. et al (2021). Evaluation of mycotoxins in infant breast milk and infant food, reviewing the literature data.	[22]
Ishikawa AT. Et al (2016). Exposure assessment of infants to Aflatoxin M_1 through consumption of breast milk and infant powdered milk in Brazil.	[60]
Juan, C. et al (2014). Presence of mycotoxin in commercial infant formulas and baby foods from Italian market.	[67]
Kabak, B. et al (2012). AFM1 and ochratoxin A in baby formulae in Turkey: Occurrence and safety evaluation.	[66]
Meucci, V. et al (2010). Mycotoxin detection in infant formula milks in Italy.	[65]
Ojuri O.T. et al (2018). Assessing the mycotoxicological risk from consumption of complementary foods by infants and young children in Nigeria.	[71]
Zinedine A. et al (2021). Aflatoxin M1 in Africa: Exposure assessment, regulations, and prevention strategies - A review.	[59]
